# The CAF1-NOT complex of trypanosomes

**DOI:** 10.3389/fgene.2013.00299

**Published:** 2014-01-02

**Authors:** Esteban Erben, Chaitali Chakraborty, Christine Clayton

**Affiliations:** Zentrum für Molekulare Biologie der Universität Heidelberg, DKFZ-ZMBH AllianceHeidelberg, Germany

**Keywords:** *Trypanosoma*, deadenylation, mRNA decay, mRNA degradation, NOT complex, CAF1

## Abstract

In African trypanosomes, there is no control of transcription initiation by RNA polymerase II at the level of individual protein-coding genes. Transcription is polycistronic, and individual mRNAs are excised by *trans*-splicing and polyadenylation. As a consequence, trypanosomes are uniquely reliant on post-transcriptional mechanisms for control of gene expression. Rates of mRNA decay vary over up to two orders of magnitude, making these organisms an excellent model system for the study of mRNA degradation processes. The trypanosome CAF1-NOT complex is simpler than that of other organisms, with no CCR4 or NOT4 homolog: it consists of CAF1, NOT1, NOT2, NOT5 NOT9, NOT10, and NOT11. It is important for the initiation of degradation of most, although not all, mRNAs. There is no homolog of NOT4, and Tho and TREX complexes are absent. Functions of the trypanosome NOT complex are therefore likely to be restricted mainly to deadenylation. Mechanisms that cause the NOT complex to deadenylate some mRNAs faster than others must exist, but have not yet been described.

## GENE EXPRESSION IN TRYPANOSOMES

African trypanosomes are protists that belong to the class Kinetoplastida. The class is characterized by a unique assemblage of mitochondrial DNA, visible by Giemsa staining, called the kinetoplast. Kinetoplastids belong to the diffuse assemblage of unicellular organisms called Excavata, which branched away from other groups, such as plants and Opisthokonts, very early in eukaryotic evolution (Rodríguez-Ezpeleta et al., 2007; Burki et al., 2008). This evolutionary position also makes it much more informative than yeasts when studying the conservation of fundamental cellular processes. The order Trypanosomatida includes several parasites of economic or medical importance, such as various Leishmanias, *Trypanosoma cruzi*, and the African trypanosomes. The disease and its treatment was recently reviewed in Barrett and Croft, 2012. The African trypanosome *T. brucei* is the most experimentally accessible member of the Excavata and has become established as a model organism for many aspects of Kinetoplastid biology.

*Trypanosoma brucei* multiplies in the blood and tissue fluids of a mammals, and within the digestive tract of Tsetse flies. In the blood the temperature is 37°C, the major substrate for energy metabolism is glucose, and the parasite must survive humoral immunity. In the Tsetse midgut, the temperature probably varies between about 20 and 39°C, the major substrates for energy metabolism are amino acids, and the parasites must survive proteases and insect innate immunity (Matthews, 2005; Bringaud et al., 2006). Immune evasion involves control of parasite surface protein expression (Stockdale et al., 2008). The dominant metabolic adaptation is a switch from glucose-based ATP generation, by glycolysis and substrate-level phosphorylation, to a system based on oxidative phosphorylation and a fully developed mitochondrion using amino acid substrates (Matthews, 2005; Bringaud et al., 2006).

The gene organization of Kinetoplastids is remarkable. Instead of having a polymerase II promoter in front of every gene, the protein-coding open reading frames (ORFs) are arranged in a head-to-tail fashion across long polycistronic transcription units (Daniels et al., 2010). The nascent transcripts are co-transcriptionally processed by *trans*-splicing of a “spliced leader (SL)” sequence of around 40 nt to the 5′ end (Michaeli, 2011). Each organism has over a hundred copies of the SL RNA genes, which – in contrast to the protein-coding regions– each have a polymerase II promoter, necessary both for regulation and to ensure the presence of a 5′ cap. The *SL* RNAs include the capped SL and an intron of around 100 nt (Michaeli, 2011). Trypanosome mRNAs are polyadenylated, but there are no polyadenylation signals within the mRNA 3′-untranslated region (3′-UTR). Instead, polyadenylation of each mRNA is coupled to *trans*-splicing of the mRNA immediately downstream. Instead of there being a single polyadenylation site, multiple sites are chosen, approximately 100 nt upstream of the polypyrimidine tract that marks the next *trans*-splicing acceptor site (Clayton and Michaeli, 2011).

The transcription units that encode the major surface proteins, are, exceptionally, transcribed by RNA polymerase I, and their transcription is epigenetically regulated (Figueiredo et al., 2009). In contrast, polymerase II polycistronic transcription units are transcribed constitutively at similar rates, and the genes within them nearly always show no relationship, either functionally or with respect to regulation (Martinez-Calvillo et al., 2010; for an exception see Kelly et al., 2012). Nevertheless, some genes are represented by hundreds of mRNAs per cell, while others are represented by one RNA, or none at all (Manful et al., 2011). Moreover, several hundred mRNAs show strong developmental regulation (Siegel et al., 2010). Many of the most strongly represented mRNAs are encoded by multiple gene copies, but beyond that, regulation has to be achieved through control of splicing and mRNA degradation. Results so far have strongly implicated mRNA degradation as a major control point. In most cases so far examined the decay rate has been found to be determined by sequences in the 3′-UTR of the mRNA (Clayton and Shapira, 2007) and interactions with RNA-binding proteins (Fernández-Moya and Estévez, 2010; Kramer and Carrington, 2011; Clayton, 2013; Droll et al., 2013).

Trypanosomes contain three different types of deadenylation complex: PAN2/PAN3 (Schwede et al., 2009), three proteins related to PARN (Utter et al., 2011), and a CAF1/NOT complex (Schwede et al., 2008). There is also an exosome (Estévez et al., 2001, 2003), and a cytosolic exoribonuclease (XRNA) responsible for 5′-3′ degradation (Li et al., 2006). Strangely, there is no detectable homolog of any of the known eukaryotic mRNA decapping enzymes (Schwede et al., 2009), although a scavenger decapping enzyme (homolog of DcpS) has been characterized (Milone et al., 2002).

Trypanosomes have an RNA interference (RNAi) machinery (Barnes et al., 2012). It is active in reducing the levels of retroposon-like RNAs (Shi et al., 2004), but it appears to have no role in regulation of expression of most genes and is not required for survival of the organisms in culture (Janzen et al., 2006). High throughput sequencing of RNAs associated with AGO1 revealed no evidence for miRNAs (Tschudi et al., 2012).

## THE COMPOSITION OF THE TRYPANOSOME CAF1/NOT COMPLEX

The CAF1/NOT complex of trypanosomes was isolated by affinity purification using CAF1 (Schwede et al., 2008) or NOT10 (Färber et al., 2013) bearing a tandemly arranged tag. This yielded homologs of human NOT1, NOT2, NOT3, NOT10, NOT11, and NOT9/CAF40. The helicase DHH1 (Kramer et al., 2010) was also present in the preparation (Färber et al., 2013). There are no convincing homologs of NOT4 or Caf130 in the available Kinetoplastid genomes. All predicted proteins similar to Ccr4 lack the leucine-rich repeat that is required for interaction with the complex, and the best match showed no interaction with the complex in a pull-down assay (Schwede et al., 2008). The trypanosome complex is therefore simpler than those of yeast and humans, and contains only a single subunit with deadenylase enzyme activity.

The CCR4/CAF1/NOT complexes of Opisthokonts have roles in transcription, some of which operate via NOT4 and the ubiquitination pathway and/or involve the Tho and TREX complexes (see other articles in this issue). Trypanosomes lack NOT4 and Tho or TREX complex subunits. Involvement of the trypanosome complex in transcription or ubiquitination therefore seems very unlikely.

Yeast two-hybrid assays with the trypanosome proteins have shown interactions between CAF1 and NOT10 and the N-terminal half of NOT1, and between NOT10 and NOT3 (Färber et al., 2013). Both NOT10 and CAF1 also weakly interacted with the C-terminal half of NOT1. We have not yet attempted any experiments with full length NOT1; NOT3 did not interact with either of the two halves (Färber et al., 2013). When NOT10 was depleted, CAF1 became detached from the complex and the level of NOT1 decreased. This suggests that in trypanosomes, NOT10 is required for the integrity of the CAF1/NOT complex. The results of similar experiments indicated, however, that this is not true in human cells (Färber et al., 2013).

**FIGURE 1:**
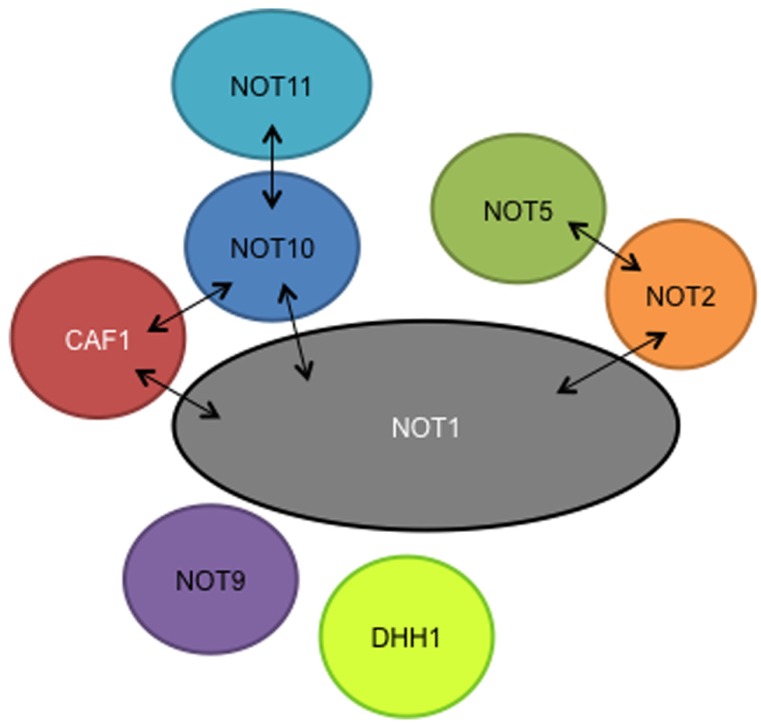
**Putative subunits of the trypanosome NOT complex, as judged by tandem affinity purification.** Association of DHH1 is unclear. Two-hybrid interactions are indicated by arrows.

The interactions of the remaining subunits have yet to be studied in detail. Our preliminary results suggest that as in yeast and animal cells, NOT2 interacts with NOT5 and the NOT1 C-terminus, and NOT11 interacts with NOT10 (**Figure 1**). So far the only evidence for CAF40/NOT9 association is from tandem affinity purification: yeast two-hybrid results have been negative. However, for NOT1 we have used only N- and C-terminal fragments (Färber et al., 2013) and it is possible that this could have precluded some interactions. We do not know whether DHH1 is specifically associated with the trypanosome CAF1/NOT complex, since we find it rather often when purifying proteins associated with mRNA metabolism and it probably has several cellular roles (Färber et al., 2013).

## ROLE OF THE CAF1/NOT COMPLEX IN TRYPANOSOME mRNA DEGRADATION

The average half-life of trypanosome total mRNA can be measured using Northern blots hybridized with a SL probe. This yields a value of about 30 min for bloodstream forms (Fadda et al., 2013). A similar estimate was made using less direct methods (Haanstra et al., 2008; Manful et al., 2011). Trypanosome poly(A) tails are up to 150 nt long. Addition of Actinomycin D to bloodstream form trypanosomes for 60 min results in a strong shift toward shorter lengths, with loss of more than 50% of poly(A) tails above 70 nt (Schwede et al., 2008). This is clear evidence for deadenylation.

Results for individual trypanosome mRNAs studied by Northern blotting have shown that half-lives can vary between about 5 min to over 4 h (Clayton and Shapira, 2007). To measure half-lives of the mRNAs from every individual ORF, we inhibited transcription and *trans*-splicing for 30 min, and, using RNASeq, compared the resulting transcriptomes with those at steady state. We assumed exponential degradation to derive a half-life for each ORF. This obviously gives only a very approximate idea of the half-life of each mRNA, since non-linear kinetics cannot be detected, but nevertheless the results for several mRNAs were acceptably similar to Northern blot values (Manful et al., 2011). Using total (rRNA-depleted) RNA for the transcriptome analysis, we found that when individual sequences were compared, the median half-life was 13 min. This assay included deadenylated transcripts, which could be decay intermediates. In contrast, using poly(A)+ mRNA, measured half-lives were on average about 9 min shorter (Manful et al., 2011). This tells us two things. First, for most mRNAs, as expected, deadenylation is the first step in degradation. Second, for an average mRNA, there is 9 min between poly(A) tail removal and destruction of the ORF.

To study the functions of the CAF1/NOT subunits, their expression was decreased by RNAi. Depletion of CAF1, NOT1 (Schwede et al., 2008), NOT3, NOT10, and CAF40 (Färber et al., 2013) all severely inhibited trypanosome growth. RNAi targeting NOT2 had no effect on growth (Färber et al., 2013), but this result must be viewed with caution since we do not know the extent to which the protein had decreased.

Results from RNAi targeting CAF1 clearly showed that CAF1 is the major deadenylase in trypanosomes. Depletion of CAF1 both increased the average length of total poly(A) tails, and delayed the decrease in their length after transcription inhibition (Schwede et al., 2008). It also caused a delay in deadenylation and decay of several constitutively expressed mRNAs, as determined by Northern blotting (Schwede et al., 2008). Interestingly, though, four extremely unstable developmentally regulated mRNAs were not so severely affected (Schwede et al., 2008). Additional experiments have shown that this last class of mRNAs can be degraded by two independent pathways. Although part of the mRNA is attacked by CAF1/NOT, a significant proportion is degraded in a deadenylation-independent fashion by the 5′–3′ exoribonuclease XRNA (Schwede et al., 2009). A similar phenomenon is seen in the related Kinetoplastid *Leishmania* (Haile et al., 2008).

Our studies of transcriptome-wide mRNA decay showed that depletion of CAF1 caused drastic inhibition. Indeed, most mRNAs appeared not to have decreased at all after 30 min transcription inhibition (Fadda et al., 2013). The only ones that seemed to buck this trend were, as before, some of the less stable mRNAs. In contrast, XRNA depletion preferentially affected unstable mRNAs (Manful et al., 2011). This is consistent with the results described above: some short-lived mRNAs are decapped then degraded by XRNA without prior deadenylation. Depletion of PAN2 or an essential exosome subunit had minor effects, mostly on mRNAs of intermediate stability. The roles of the PARN proteins are unclear (Utter et al., 2011).

## THE NOT COMPLEX IS NEEDED TO RECRUIT CAF1 TO ITS mRNA SUBSTRATES

Depletion of CNOT10 completely inhibited deadenylation-dependent mRNA decay (Fadda et al., 2013). Since we also knew that NOT10 depletion resulted in detachment of CAF1 from the NOT complex (Färber et al., 2013), but isolated CAF1 has enzyme activity (Schwede et al., 2008), the obvious interpretation is that recruitment of CAF1 to mRNAs depends on interactions with other components of the complex. To test this hypothesis further, we expressed CAF1 with an N-terminal lambda N peptide. In the same cells, we also expressed a reporter mRNA bearing five copies of the lambda N recognition sequence, boxB. The co-expression was expected to result in “tethering” of the lambda-N-CAF1 fusion to the reporter bearing box B via the lambdaN-boxB interaction, Indeed, the boxB-containing reporter was completely destroyed by co-expression of LambdaN-CAF1. In contrast, and tethering of lambdaN-GFP increased expression by about 10%, and a reporter without the boxB sequence was unaffected by LambdaN-CAF1 (Färber et al., 2013). Together, these results indicate that *in vivo*, CAF1 needs to be actively recruited to mRNAs in order to degrade them. Preliminary results from a high-throughput tethering screen suggest that several other CAF1/NOT subunits are also able to promote mRNA degradation – presumably by recruiting the rest of the complex (E. Erben, manuscript in preparation). Similar tethering results have been described for the subunits of the *Drosophila* NOT complex (Bawankar et al., 2013).

If all trypanosome mRNAs are produced constitutively, and relative abundances are determined only by mRNA decay, then inhibition of the decay should cause the abundances of all mRNAs to even out. However, this does not happen. Instead, RNAi targeting either CAF1 or NOT10 caused relatively minor changes in mRNA abundance (Färber et al., 2013), with increases only in a small number of RNAs. This suggests that a feedback mechanism prevents total disruption of mRNA control: control of splicing, or slower transcription, are possibilities. Nevertheless, the mechanism clearly breaks down quite fast since the cells are unable to survive.

It is known for all organisms that NOT1 is a central scaffold of the complex. We therefore postulate that the other subunits – NOT2, NOT3, NOT10, NOT11, and CAF40 – may have roles in recruiting CAF1/NOT to specific mRNAs. Presumably, mRNAs that undergo rapid deadenylation associate with sequence-specific RNA-binding proteins which, in turn, are able to interact with complex components. We are currently searching for additional candidates using yeast two-hybrid screens.

## Conflict of Interest Statement

The authors declare that the research was conducted in the absence of any commercial or financial relationships that could be construed as a potential conflict of interest.
